# Supplementary figures and images for: Implications of tissue specific STING protein flux and abundance on inflammation and the development of targeted therapeutics
Source: PLoS One. 2025 Feb 25;20(2):e0319216. doi: 10.1371/journal.pone.0319216 (PMC11856325; doi:10.1371/journal.pone.0319216)

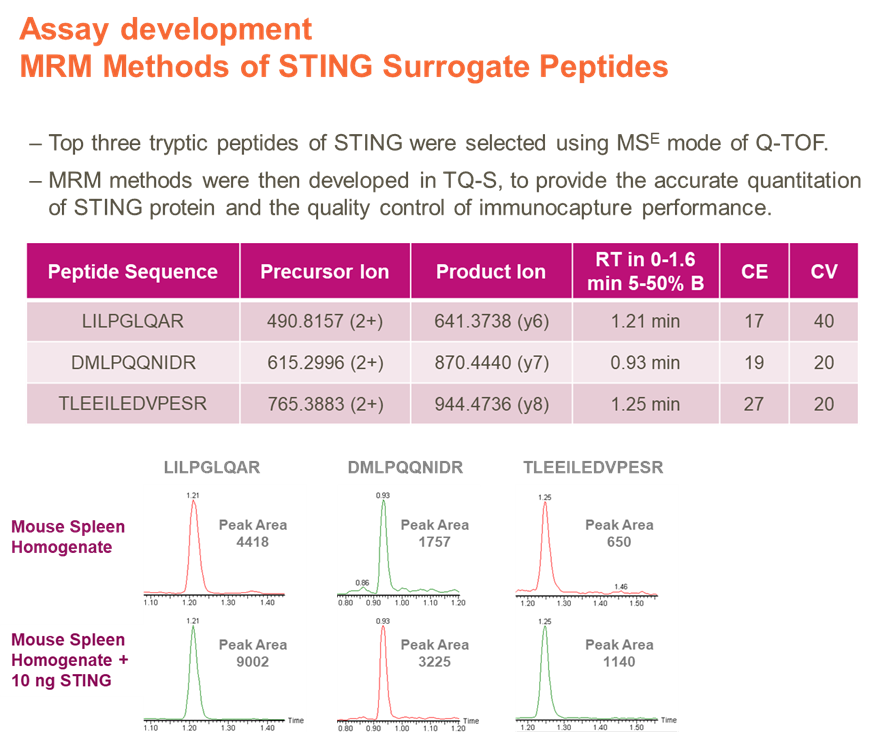

Supplement: S2 Fig — (TIF) [file pone.0319216.s002.tif]
