# Supplementary material for: Implications of tissue specific STING protein flux and abundance on inflammation and the development of targeted therapeutics
Source: PLoS One. 2025 Feb 25;20(2):e0319216. doi: 10.1371/journal.pone.0319216 (PMC11856325; doi:10.1371/journal.pone.0319216)
Supplement: Attachment — Submitted filename: Response to Reviewers.docx [file pone.0319216.s005.docx]

COPY OF REVISION DECISION LETTER WITH ADDED RESPONSES

**View Letter**

| **Date:** | Jun 12 2024 07:31PM |
| --- | --- |
| **To:** | "G Scott Pesiridis" g.s.pesiridis@gsk.com |
| **From:** | "PLOS ONE" plosone@plos.org |
| **Subject:** | PLOS ONE Decision: Revision required [PONE-D-24-15873] |

PONE-D-24-15873

Implications of tissue specific STING protein flux and abundance on the development of therapeutics.

PLOS ONE

Dear Dr. Pesiridis,

Thank you for submitting your manuscript to PLOS ONE. After careful consideration, we feel that it has merit but does not fully meet PLOS ONE’s publication criteria as it currently stands. Therefore, we invite you to submit a revised version of the manuscript that addresses the points raised during the review process. Specifically, both reviewers have provided a well thought-out critic of the manuscript and have commented on background and foundational aspects of the study that need to be addressed as appropriate.

Please submit your revised manuscript by Jul 27 2024 11:59PM. If you will need more time than this to complete your revisions, please reply to this message or contact the journal office at [plosone@plos.org](mailto:plosone@plos.org). When you're ready to submit your revision, log on to https://www.editorialmanager.com/pone/ and select the 'Submissions Needing Revision' folder to locate your manuscript file.

Please include the following items when submitting your revised manuscript:

- A rebuttal letter that responds to each point raised by the academic editor and reviewer(s). You should upload this letter as a separate file labeled 'Response to Reviewers'.
- A marked-up copy of your manuscript that highlights changes made to the original version. You should upload this as a separate file labeled 'Revised Manuscript with Track Changes'.
- An unmarked version of your revised paper without tracked changes. You should upload this as a separate file labeled 'Manuscript'.

If you would like to make changes to your financial disclosure, please include your updated statement in your cover letter. Guidelines for resubmitting your figure files are available below the reviewer comments at the end of this letter.

If applicable, we recommend that you deposit your laboratory protocols in protocols.io to enhance the reproducibility of your results. Protocols.io assigns your protocol its own identifier (DOI) so that it can be cited independently in the future. For instructions see: <https://journals.plos.org/plosone/s/submission-guidelines#loc-laboratory-protocols>. Additionally, PLOS ONE offers an option for publishing peer-reviewed Lab Protocol articles, which describe protocols hosted on protocols.io. Read more information on sharing protocols at <https://plos.org/protocols?utm_medium=editorial-email&utm_source=authorletters&utm_campaign=protocols>.

We look forward to receiving your revised manuscript.

Kind regards,

Jon M. Jacobs, Ph.D.
Academic Editor
PLOS ONE

Journal requirements:

When submitting your revision, we need you to address these additional requirements.

1. Please ensure that your manuscript meets PLOS ONE's style requirements, including those for file naming. The PLOS ONE style templates can be found at

https://journals.plos.org/plosone/s/file?id=wjVg/PLOSOne_formatting_sample_main_body.pdf and

<https://journals.plos.org/plosone/s/file?id=ba62/PLOSOne_formatting_sample_title_authors_affiliations.pdf>

Major edits and formatting are complete to meet the style requirements as proposed in the revised version of the manuscript and figures. In particular, changes were made to provide a separate title page with suggested formats, align Headings and Subheadings, figure references, placement of figure legends, font and citation styles, tables, figures and file naming standards. Please let me know if I’ve missed something.

2. We noticed you have some minor occurrence of overlapping text with the following previous publication(s), which needs to be addressed:

-  Improved Sensitivity for Protein Turnover Quantification by Monitoring Immonium Ion Isotopologue Abundance - https://doi.org/10.1021/acs.analchem.9b01329.

(Among others)

In your revision ensure you cite all your sources (including your own works), and quote or rephrase any duplicated text outside the methods section. Further consideration is dependent on these concerns being addressed.

The introduction was edited to rephrase text with some minor occurrence of overlapping text from the lead author’s previous publication cited above.

3. We note that the grant information you provided in the ‘Funding Information’ and ‘Financial Disclosure’ sections do not match.

When you resubmit, please ensure that you provide the correct grant numbers for the awards you received for your study in the ‘Funding Information’ section.

All funding for the studies was provided by GSK. There are no grant numbers to reference and have made more explicit statement in the ‘Funding Information’ section.

4. Thank you for stating the following in the Competing Interests section:

“All authors listed are current or former employees of GSK with shares in GSK plc.”

Please confirm that this does not alter your adherence to all PLOS ONE policies on sharing data and materials, by including the following statement: "This does not alter our adherence to  PLOS ONE policies on sharing data and materials.” (as detailed online in our guide for authors http://journals.plos.org/plosone/s/competing-interests).  If there are restrictions on sharing of data and/or materials, please state these. Please note that we cannot proceed with consideration of your article until this information has been declared.

Please include your updated Competing Interests statement in your cover letter; we will change the online submission form on your behalf.

Please use the revised Competing Interests statement as suggested; “All authors listed are current or former employees of GSK who have owned shares in the past or currently hold shares in GSK plc. This does not alter our adherence to PLOS ONE policies on sharing data and materials.”

5. We note that your Data Availability Statement is currently as follows: [All relevant data are within the manuscript and its Supporting Information files.]

Please confirm at this time whether or not your submission contains all raw data required to replicate the results of your study. Authors must share the “minimal data set” for their submission. PLOS defines the minimal data set to consist of the data required to replicate all study findings reported in the article, as well as related metadata and methods (https://journals.plos.org/plosone/s/data-availability#loc-minimal-data-set-definition).

For example, authors should submit the following data:

- The values behind the means, standard deviations and other measures reported;

- The values used to build graphs;

- The points extracted from images for analysis.

Authors do not need to submit their entire data set if only a portion of the data was used in the reported study.

If your submission does not contain these data, please either upload them as Supporting Information files or deposit them to a stable, public repository and provide us with the relevant URLs, DOIs, or accession numbers. For a list of recommended repositories, please see https://journals.plos.org/plosone/s/recommended-repositories.

If there are ethical or legal restrictions on sharing a de-identified data set, please explain them in detail (e.g., data contain potentially sensitive information, data are owned by a third-party organization, etc.) and who has imposed them (e.g., an ethics committee). Please also provide contact information for a data access committee, ethics committee, or other institutional body to which data requests may be sent. If data are owned by a third party, please indicate how others may request data access.’

Yes, I confirm that all relevant data are within the manuscript and its Supporting Information files.

6. Please amend either the title on the online submission form (via Edit Submission) or the title in the manuscript so that they are identical.

The titles now agree.

7. We note that S1 Fig 2 in your submission contain copyrighted images. All PLOS content is published under the Creative Commons Attribution License (CC BY 4.0), which means that the manuscript, images, and Supporting Information files will be freely available online, and any third party is permitted to access, download, copy, distribute, and use these materials in any way, even commercially, with proper attribution. For more information, see our copyright guidelines: http://journals.plos.org/plosone/s/licenses-and-copyright.

We require you to either (1) present written permission from the copyright holder to publish these figures specifically under the CC BY 4.0 license, or (2) remove the figures from your submission:

I confirm that the aforementioned figure and has been removed.

1. You may seek permission from the original copyright holder of S1 Fig 2 to publish the content specifically under the CC BY 4.0 license.

We recommend that you contact the original copyright holder with the Content Permission Form (http://journals.plos.org/plosone/s/file?id=7c09/content-permission-form.pdf) and the following text:

“I request permission for the open-access journal PLOS ONE to publish XXX under the Creative Commons Attribution License (CCAL) CC BY 4.0 (http://creativecommons.org/licenses/by/4.0/). Please be aware that this license allows unrestricted use and distribution, even commercially, by third parties. Please reply and provide explicit written permission to publish XXX under a CC BY license and complete the attached form.”

Please upload the completed Content Permission Form or other proof of granted permissions as an "Other" file with your submission.

In the figure caption of the copyrighted figure, please include the following text: “Reprinted from [ref] under a CC BY license, with permission from [name of publisher], original copyright [original copyright year].”

2. If you are unable to obtain permission from the original copyright holder to publish these figures under the CC BY 4.0 license or if the copyright holder’s requirements are incompatible with the CC BY 4.0 license, please either i) remove the figure or ii) supply a replacement figure that complies with the CC BY 4.0 license. Please check copyright information on all replacement figures and update the figure caption with source information. If applicable, please specify in the figure caption text when a figure is similar but not identical to the original image and is therefore for illustrative purposes only.

8. We notice that your supplementary figures are included in the manuscript file. Please remove them and upload them with the file type 'Supporting Information'. Please ensure that each Supporting Information file has a legend listed in the manuscript after the references list.

[Note: HTML markup is below. Please do not edit.]

Reviewers' comments:

Reviewer's Responses to Questions

**Comments to the Author**

1. Is the manuscript technically sound, and do the data support the conclusions?

The manuscript must describe a technically sound piece of scientific research with data that supports the conclusions. Experiments must have been conducted rigorously, with appropriate controls, replication, and sample sizes. The conclusions must be drawn appropriately based on the data presented.

Reviewer #1: No

Reviewer #2: Yes

2. Has the statistical analysis been performed appropriately and rigorously?

Reviewer #1: Yes

Reviewer #2: Yes

3. Have the authors made all data underlying the findings in their manuscript fully available?

The [PLOS Data policy](http://www.plosone.org/static/policies.action#sharing) requires authors to make all data underlying the findings described in their manuscript fully available without restriction, with rare exception (please refer to the Data Availability Statement in the manuscript PDF file). The data should be provided as part of the manuscript or its supporting information, or deposited to a public repository. For example, in addition to summary statistics, the data points behind means, medians and variance measures should be available. If there are restrictions on publicly sharing data—e.g. participant privacy or use of data from a third party—those must be specified.

Reviewer #1: Yes

Reviewer #2: No

4. Is the manuscript presented in an intelligible fashion and written in standard English?

PLOS ONE does not copyedit accepted manuscripts, so the language in submitted articles must be clear, correct, and unambiguous. Any typographical or grammatical errors should be corrected at revision, so please note any specific errors here.

Reviewer #1: Yes

Reviewer #2: Yes

5. Review Comments to the Author

Please use the space provided to explain your answers to the questions above. You may also include additional comments for the author, including concerns about dual publication, research ethics, or publication ethics. (Please upload your review as an attachment if it exceeds 20,000 characters)

Reviewer #1: In this manuscript, Thomas Angel et al. use 2H2O to measure protein fractional synthesis rates (FSR) of STING across different tissues in vivo. The authors reached a conclusion that the FSR of STING varies from 0.027 in muscle to 0.731 in lung per day, and the half-life of STING varies from 3.8 to 25.4 days. Even unexpectedly, the half-life of STING in WT mice and STING-driven auto-immune TREX1D18N/D18N mice are largely comparable. Though the authors used cutting-edge techniques in the experiments, the conclusion is still not fully convincing, mainly due to very limited data provided and one single approach used.

1. The authors show the half-life of activated STING is 2 hours in Fig 1a, and this is consistent with most previous studies. The authors should use this widely-used system to compare with 2H2O-based techniques used in this study to verify the latter approach indeed works.

The loss of STING upon stimulation in cell systems and in whole tumors shown in Fig 1 is a consequence of pathway stimulation and not a steady state turnover of the protein under sterile or unstimulated conditions. As noted, the point made in Fig 1 is in agreement with published reports (Gonugunta VK, Cell reports. 2017) and only characterizes the complete degradation of STING rather than the turnover which considers both resynthesis and degradation. We have added comments to the discussion to compare different methods to measure protein half-life along with limitations of classical methods that employ protein synthesis inhibitors *in vitro*. We also cite whole cell proteomics research in primary human cells that estimate the protein half-life to be on the order of hundreds of hours depending on the cell type (> 500 hours in primary human monocytes) using the approach published by Mathieson T, et al Systematic analysis of protein turnover in primary cells. Nat Commun. 2018;9(1):689.

The 2H2O approach used in this paper is a well-established and recognized method for the quantitative assessment of protein flux, both loss of protein and resynthesis, in vivo.  This includes methods validated in rodents, monkey and man.

1) Shankaran, M., King, C. L., Angel, T. E., Holmes, W. E., Li, K. W., Colangelo, M., ... & Hellerstein, M. K. (2016). Circulating protein synthesis rates reveal skeletal muscle proteome dynamics. *The Journal of clinical investigation*, *126*(1), 288-302.
2) Gasier, H. G., Fluckey, J. D., & Previs, S. F. (2010). The application of 2 H 2 O to measure skeletal muscle protein synthesis. *Nutrition & metabolism*, *7*, 1-8.,
3) Miller, B. F., Reid, J. J., Price, J. C., Lin, H. J. L., Atherton, P. J., & Smith, K. (2020). CORP: The use of deuterated water for the measurement of protein synthesis. *Journal of Applied Physiology*, *128* (5), 1163-1176.
4) Holwerda, A. M., Atherton, P. J., Smith, K., Wilkinson, D. J., Phillips, S. M., & van Loon, L. J. (2024). Assessing muscle protein synthesis rates in vivo in humans: the deuterated water (2H2O) method. *The Journal of Nutrition*.
5) Holmes, W. E., Angel, T. E., Li, K. W., & Hellerstein, M. K. (2015). Dynamic proteomics: in vivo proteome-wide measurement of protein kinetics using metabolic labeling. *Methods in enzymology*, *561*, 219-276.
6) Angel, T. E., Naylor, B. C., Price, J. C., Evans, C., & Szapacs, M. (2019). Improved sensitivity for protein turnover quantification by monitoring Immonium ion Isotopologue abundance. *Analytical chemistry*, *91*(15), 9732-9740.
7) Fu, X., Deja, S., Fletcher, J. A., Anderson, N. N., Mizerska, M., Vale, G., ... & Burgess, S. C. (2021). Measurement of lipogenic flux by deuterium resolved mass spectrometry. *Nature communications*, *12*(1), 3756.

2. The authors also need more supportive data to verify their approach. For example, comparing their 2H2O-based techniques with standard CHX chase experiments to further validate their study. CHX chase experiments can at least be done in vitro using some culturable tissue or cells from WT and TREX1D18N/D18N mice.

Processes that use transcription or translation inhibitors along with pulse-chase labeling approaches are limited to cellular systems and on a time-scale that is on the order of minutes and hours rather than days (which is required for accurate measures of STING protein in vivo).  Furthermore, protein half-life measured by CHX pulse-chase approaches only capture the loss of protein, but fail to capture the tissue specific protein synthesis. Application of cycloheximide creates an artificial, non-biologically relevant, non equilibrium state for cells in culture and does not represent normal in vivo biology. Application of deuterium oxide at the levels used for metabolic labeling does not change the basal state of the organism allowing normal protein synthesis and degradation to occur over the time interval where protein dynamics are being measured. Please see Chan C, et al. Incompatibility of chemical protein synthesis inhibitors with accurate measurement of extended protein degradation rates. Pharmacol Res Perspect. 2017;5(5).

3. The authors conclude the half-life of STING is at least 3.8 days, which is quite longer than the observed static STING half-life from CHX chase experiments in previous literature. Method-wise, mice were injected with 2H2O once followed by enriched drinking water (8% 2H2O) for the remainder of the experiment. Is this approach sufficient to make any amino acids in the mice incorporated with 2H2O during the whole experiment? If only a fraction of amino acids in the cell is incorporated with 2H2O and the unlabeled amino acids, for example, recycled from digested protein by the proteasome, can still be used in synthesizing STING, then the techniques used cannot detect the actual synthesis rate of STING, resulting in lower than the actual rate. This needs to be thoroughly addressed.

Half-life of STING in tissues when measured in an intact animal is different than observed in immortalized cells or primary cells in culture. This is not unexpected as the metabolic rates of immortalized cells and primary cells in culture is very different than in the whole intact animal and illustrates that context matters. A fundamental finding of our research is that the turnover rate is indeed different in different environments and hence needs to be accurately measured in the system we use to describe the biology or treat with therapeutic modalities. The in vitro system cited by this reviewer will simply not work in vivo and is quite toxic over long periods of time.

Deuterium oxide is a convenient and universal stable isotope labeling reagent, given that it can be consumed orally, is nontoxic, rapidly equilibrates with the total body water in about 30 minutes, enabling the labeling of a wide range of biopolymers including nucleotides, lipids, and proteins. Hydrogen atoms from body water are incorporated into free amino acids through de novo biosynthesis. The labeled pool of free amino acids rapidly reaches equilibrium with body water enrichment (10-20 min) in rodent.  Belloto, E., Diraison, F., Basset, A., Allain, G., Abdallah, P., & Beylot, M. (2007). Determination of protein replacement rates by deuterated water: validation of underlying assumptions. *American Journal of Physiology-Endocrinology and Metabolism*, *292*(5), E1340-E1347.

Reviewer #2: In this manuscript from researchers at GlaxoSmithKline, the authors document the protein turn-over of STING in wildtype (WT) C57Bl/6 mice and those carrying the Acardi-Goutiéres syndrome (AGS)-associated mutant isoform of Trex1 (D18N homozygous). The authors demonstrate that STING protein half-life is tissue specific and similar between WT and AGS mice, with the shortest half-life in colon and lymph node and longest in skeletal muscle. Overall, the experiments are well executed, and this study constitutes important but rather specialist information that will be useful to those researchers working on STING drug development.


Major Comments

1) The authors need to explicitly state where they obtained the Trex1 [D18N/D18N] model, as the details are not clear in the methods. Was this generated by the authors, did they receive it from a laboratory? What specific model is it? The original Trex1[D18N/D18N] model was in the 129S6/SvEvTac mouse background, not C57Bl/6. Did the authors obtain the mice already in the C57Bl/6 background, or did they do the backcrossed themselves. Really, much more detail is needed here to ensure future reproducibility and establish the provenance of the mice.

We have edited the manuscript to be more explicit about the origin of the animals used in our studies. Briefly, these animals were generation in a fee-for-service collaboration with GenoWay and are owned by GSK. The official name of the strain is C57BL/6NCrl-*Trex1^tm1.1(D18N)Geno^*/Gsk. We added the details of their generation including an additional paragraph in the Materials and Methods, a paragraph in the Results section, and a new supplemental figure (S1 Fig) with pertinent characterization data. Furthermore, we added a citation where additional characterization data can be found in our collaboration with Humphries F, Shmuel-Galia L, Jiang Z, Zhou JY, Barasa L, Mondal S, et al. Targeting STING oligomerization with small-molecule inhibitors. Proc Natl Acad Sci U S A. 2023;120(33):e2305420120.

2) This is a rather limited manuscript in scope, and of VERY specialist interest. To move beyond specialist interest for drug development, the authors should provide correlation data on interferon stimulated gene (ISG) regulation down-stream of STING relative to protein turn-over and abundance in the tissues tested and between WT and AGS mice. This could be done using a basket of ISGs and a qPCR panel, or if budget allows via RNA-seq. This information would greatly increase the impact of the current study and interest for a broader range of researchers and in particular immunologists.

Data and correlations of ISG transcription are now included in the revised manuscript. We appreciate the feedback on this point since the data really captures added depth to how tissue specific inflammation is related to STING protein abundance and turnover.

Minor Comments

1) Despite the misuse of human gene and protein (ALL CAPS) nomenclature by the original creators of the Trex1[D18N/D18N] mouse – the authors throughout the manuscript should use the correct gene and protein nomenclature for the mouse Trex1, which is capitalized first letter and then lowercase.

A corrected nomenclature is now included and references as Trex1^D18N^ as a shortened form of C57BL/6NCrl-*Trex1^tm1.1(D18N)Geno^*/Gsk. It is explicitly stated in the text and we are open to an alternative short name.

2) In the Author contributions please clarify what “…in-life TREX [D18N/D18N]…” means?

This refers to the in-life portion of the animal study including necropsy. I’ve edited the statement to “animal studies and necropsy.” Please let me know if this is adequate.

6. PLOS authors have the option to publish the peer review history of their article ([what does this mean?](https://journals.plos.org/plosone/s/editorial-and-peer-review-process#loc-peer-review-history)). If published, this will include your full peer review and any attached files.


If you choose “no”, your identity will remain anonymous but your review may still be made public.

**Do you want your identity to be public for this peer review?** For information about this choice, including consent withdrawal, please see our [Privacy Policy](https://www.plos.org/privacy-policy).

Reviewer #1: No

Reviewer #2: No

[NOTE: If reviewer comments were submitted as an attachment file, they will be attached to this email and accessible via the submission site. Please log into your account, locate the manuscript record, and check for the action link "View Attachments". If this link does not appear, there are no attachment files.]

While revising your submission, please upload your figure files to the Preflight Analysis and Conversion Engine (PACE) digital diagnostic tool, <https://pacev2.apexcovantage.com/>. PACE helps ensure that figures meet PLOS requirements. To use PACE, you must first register as a user. Registration is free. Then, login and navigate to the UPLOAD tab, where you will find detailed instructions on how to use the tool. If you encounter any issues or have any questions when using PACE, please email PLOS at [figures@plos.org](mailto:figures@plos.org). Please note that Supporting Information files do not need this step.

*In compliance with data protection regulations, you may request that we remove your personal registration details at any time.* [*(Remove my information/details)*](https://www.editorialmanager.com/pone/login.asp?a=r)*. Please contact the publication office if you have any questions.*
